# Health infrastructure under pressure: spatial-temporal analysis of King Saud Medical City expansion

**DOI:** 10.3389/frhs.2026.1767964

**Published:** 2026-04-10

**Authors:** Hala A. Alosaimi, Zainab Al-Mughassil, Alfayo Omayio, Jose M. Valderas, Hasan Alghamdi

**Affiliations:** 1 King Saud Medical City, Riyadh First Health Cluster, Riyadh, Saudi Arabia; 2 Ministry of Health, Riyadh, Saudi Arabia; 3 Department of Geomatic Engineering and Geospatial Information Systems, JKUAT, Nairobi, Keyna; 4 Centre for Research in Health System Performance (CRiHSP), Yong Loo Lin School of Medicine, National University of Singapore, Singapore

**Keywords:** health planning, health system, King Saud Medical City, policy, Saudi arabia

## Abstract

King Saud Medical City (KSMC), Riyadh's largest public medical city, has undergone a profound spatial and institutional transformation between 2000 and 2025. Using multi-temporal remote sensing and geographic information system analysis, this study provides quantitative evidence of medical city densification in Saudi Arabia. The built-up footprint expanded by more than 19 hectares, and the Normalized Difference Built-up Index increased by +0.36, reflecting a shift from a low-rise, dispersed campus to a vertically integrated medical complex. This physical transformation coincided with major institutional growth: total bed capacity increased from approximately 450 to over 1,500, including 200 intensive care beds; the emergency department expanded to 102 beds, becoming one of the largest in the Kingdom; and annual surgical volume exceeded 20,000 major procedures. These changes occurred alongside rapid population growth in Riyadh and national health system reforms such as the Medical Cities Law, the Health Cluster Model, and Vision 2030. The findings demonstrate how spatial densification enabled operational expansion and accelerated the adoption of advanced medical infrastructure. The study offers a scalable spatial framework for guiding the future development of large medical facilities in rapidly urbanizing settings.

## 1 Introduction

The design and development of healthcare facilities play a crucial role in shaping the accessibility and efficiency of healthcare systems. Healthcare infrastructure is a cornerstone of community resilience, impacting not only the health outcomes of the population but also social and economic development. Globally, the expansion of hospitals is closely linked to demographic growth and disease burden patterns (1, 2). Large medical complexes have become central to urban health systems, even influencing land use and transportation, and serving as centers for education, innovation, and research (3). Therefore, the spatial and temporal developments of healthcare infrastructure extend beyond the health sector, affecting urban planning, sustainability, and policy formulation. Therefore, the urgent need to invest in health infrastructure has increased due to population growth and the prevalence of diseases, particularly in rapidly urbanizing cities. The United Nations projects that nearly 70% of the world's population will reside in cities by 2050, intensifying the demand for urban health services that are sustainable, equitable, and resilient (4). Without parallel expansion in healthcare infrastructure, rapidly urbanizing regions risk fragmented service delivery and spatial inequities, exacerbating health disparities (5). Saudi Arabia provides a compelling case in this regard. The Kingdom's population has more than doubled since the early 1990s, accompanied by rising life expectancy, rapid urban expansion, and a shift toward chronic disease prevalence (6). Riyadh, as both the capital and the largest metropolis, embodies these pressures, experiencing exponential population growth that has imposed severe stress on existing healthcare facilities (7). In response, Saudi health policy has undergone transformative reforms to ensure equitable access, efficiency, and sustainability, most prominently through Vision 2030, which emphasizes digitalization, clustering, and system-wide modernization (8).

At the center of this transformation lies King Saud Medical City (KSMC), officially recognized by the Ministry of Health as Riyadh's first modern hospital, founded in 1956 (9). Over seven decades, KSMC has grown from a medium-sized facility into the one of the Kingdom's largest medical city integrating specialized centers, high-rise inpatient towers, and state-of-the-art trauma and surgical services. Despite the importance of the long-standing history of King Saud Medical City, it has received little attention in documenting its spatial developments. Existing studies on healthcare in Saudi Arabia have focused largely on financing, service delivery, and accessibility (6, 10), with limited geospatial analysis examining how large medical cities physically evolve within mature urban environments and how such spatial transformations align with health system expansion and policy reforms. This constitutes a critical gap. The built form of hospitals encodes institutional priorities, reflects capacity constraints, and shapes the ability of health systems to adapt to urban growth. Remote sensing and geographic information systems (GIS) offer a powerful methodological avenue to fill this gap, providing longitudinal, evidence-based analysis of land-use transitions, built-up density, and urban integration of healthcare facilities (11, 12). International examples illustrate this potential: spatial analyses of Singapore General Hospital and St. Luke's International Hospital in Tokyo demonstrated how vertical densification strategies supported service efficiency in land-scarce contexts (13, 14). Yet no comparable empirical work has been undertaken for Saudi Arabia.

This study is guided by a central set of research questions that explore how King Saud Medical City (KSMC) has physically transformed over the past twenty-five years. This study focuses on the spatial and temporal patterns of urban expansion between 2000 and 2025, revealing what these patterns disclose about the hospital's development and how the changes in land use from undeveloped and vegetated land to built medical infrastructure reflect the institutional and functional growth dynamics of King Saud Medical City. Furthermore, exploring the lessons learned from this long-term spatial transformation to guide a more sustainable approach to the planning and management of large specialty hospitals in Saudi Arabia.

## 2 Materials and methods

### 2.1 Study site

King Saud Medical City, located in Riyadh, one of Saudi Arabia's largest medical city and a strategic hub within the national health system. Founded in 1956, the facility has expanded over several phases and maintains a long institutional history. King Saud Medical City was selected as a case study not only for its central role within Saudi Arabia's healthcare transformation under Vision 2030, but also for its distinctive historical and urban significance. Established in the mid-twentieth century as one of the Kingdom's earliest modern medical complexes, it occupies a rare position within an already consolidated urban fabric, unlike many newly developed medical cities built on peripheral or greenfield sites. Over decades, the medical city has undergone successive phases of operational transformation, spatial expansion, and service diversification, leaving behind a layered physical and institutional record of change. This accumulated history of growth and adaptation renders King Saud Medical City a uniquely suitable case through which to examine the interplay between healthcare infrastructure, population pressures, and evolving health policies within a mature urban context. The study boundary included the hospital complex. A retrospective space-time design was employed to examine the spatial and functional evolution of King Saud Medical City between the years 2000 and 2025. A mixed-methods geospatial expert-system approach was adopted, integrating satellite remote sensing, GIS-based classification, and historical health infrastructure data. This combination enabled a dual focus on quantitative land use changes and their relevance for growth and policy planning.

### 2.2 Time-node justification

The temporal nodes (2000, 2015, and 2025) were selected not solely based on satellite data availability, but to reflect distinct phases in institutional and national health system development. Although continuous satellite records exist, data availability alone was not considered sufficient justification for temporal selection. The year 2000 represents a pre-reform baseline before major structural modernization of the Saudi health sector. The year 2015 marks a transitional period preceding the launch of Vision 2030 and the restructuring of healthcare governance under the health cluster model. The year 2025 reflects the consolidation phase of Vision 2030 implementation, during which infrastructure scaling and vertical densification were operationalized. This phased approach enables spatial change to be interpreted within identifiable policy milestones. The temporal segmentation was conceptually anchored to identifiable governance and structural reform phases within the Saudi health system rather than solely to satellite data availability. Although continuous imagery exists, the selected nodes correspond to (i) a pre-cluster institutional phase (2000), (ii) a pre-Vision 2030 transitional restructuring period (2015), and (iii) a consolidation phase under the Health Transformation Program (2025). This alignment allows spatial change to be interpreted within institutional reform cycles rather than as isolated physical transformations.

### 2.3 Data sources

Satellite imagery. Multi-temporal Landsat datasets were obtained from the US Geological Survey: Landsat 7 ETM + (2000), Landsat 8 OLI/TIRS (2015), and Landsat 9 OLI/TIRS (2025). Sentinel-2 data from the Copernicus Open Access Hub were used as supplementary sources to ensure consistency and reduce cloud cover (<30%).

Ancillary health records. Archival materials from the Ministry of Health (MOH), KSMC's official records, and reports in Al-Jazirah newspaper were reviewed to track changes in hospital bed numbers, operating theaters, and modernization milestones. Data on capacity expansion were aligned with the three temporal nodes (2000, 2015, 2025) to allow direct comparison with geospatial outputs. Historical records were triangulated across Ministry of Health publications, hospital reports, and independent newspaper archives to minimise single-source reporting bias. Where discrepancies were detected, majority consistency and institutional validation were prioritized. Nonetheless, retrospective reporting inconsistencies cannot be fully excluded.

### 2.4 Demographic context

Population statistics for Riyadh were obtained from the General Authority for Statistics (GaStat) and international datasets (United Nations, 2019) to contextualize infrastructure demand.

### 2.5 Remote sensing and GIS processing

Pre-processed images were subjected to supervised classification using the Maximum Likelihood algorithm. Three land-cover categories were defined: built-up, bare land, and vegetation. Training samples (Regions of Interest, ROIs) were selected from visually interpretable areas in each temporal image, cross-validated with high-resolution Google Earth snapshots. Post-classification filtering was applied to reduce pixel-level noise.

Classification accuracy was evaluated using confusion matrices, overall accuracy, and the Kappa coefficient, all of which exceeded 0.89 across the three years. Ground validation was limited but supported through visual inspection against contemporaneous municipal maps and high-resolution imagery.

To mitigate cross-sensor discrepancies, all datasets were atmospherically corrected and converted to surface reflectance prior to classification. Sentinel-2 imagery (10 m) was resampled to 30 m to match the spatial resolution of Landsat imagery, ensuring comparability across time nodes. Spectral band harmonization was conducted by selecting equivalent spectral ranges (NIR and SWIR bands) to standardize index calculations. While minor radiometric differences may persist, consistency checks and cross-validation with high resolution imagery reduced classification bias.

### 2.6 Change detection

Analysis was conducted to identify shifts between land use categories across three time periods: 2000–2015, 2015–2025, and 2000–2025. The Normalized Difference Built-up Index (NDBI) was calculated to quantify the intensity and concentration of urban development. Spatial overlay techniques were used to highlight the expansion, particularly in the central and southeastern sections of the campus where new medical towers were constructed.

### 2.7 Integration with health infrastructure data

Geospatial results were compared with temporal data on KSMC's bed capacity, ICU units, operating rooms, and emergency department size. This integration enabled interpretation of land-use transitions in light of healthcare demand and institutional growth. For example, increases in built-up area were examined alongside expansions in inpatient capacity and surgical throughput.

### 2.8 Analytical framework

The analysis was guided by two frames:

First, Spatio-temporal growth detection —quantifying built-up expansion, vegetation loss, and bare land conversion. Second, policy interpretation linking observed physical changes to national health targets (e.g., Saudi Vision 2030, WHO principles for sustainable hospital design). GIS outputs were conceptualized not merely as technical findings but as decision-support evidence for health policymakers.

### 2.9 Limitations

This study has limitations in two aspects. First, the 30 m spatial resolution of Landsat imagery constrains the detection of small-scale facilities and subtle intra-campus changes within the medical city. Second, although the classification accuracy was consistently high, field verification relied mainly on secondary data sources due to the limited availability of direct field verification. Despite these limitations, the integration of multi-temporal remote sensing imagery with official records provides a robust basis for assessing long-term spatial and institutional trends. Future research should incorporate sub-meter imagery and systematic on-site validation to enhance spatial granularity and generate confidence intervals for area estimates. Although multiple validation and triangulation procedures were implemented, residual biases may persist due to sensor resolution differences, potential historical reporting inconsistencies, and the inherent constraints of retrospective analysis. Therefore, results should be interpreted as evidence of spatial institutional alignment rather than definitive causal proof. The exclusion of NDBI analysis for the year 2000 introduces a limitation in direct three-point index comparison. However, the coarse spatial resolution of Landsat 7 (30 m) relative to the small study area would have produced unreliable built-up intensity gradients. Therefore, index-based intensity analysis was initiated from 2015 using higher-resolution Sentinel data. This methodological decision prioritised analytical reliability over temporal completeness.

### 2.10 Ethical considerations

No patient-level or personally identifiable data was used. Institutional records and demographic statistics were publicly available or obtained from official government sources. No formal ethical approval was required.

## 3 Results

Accuracy assessment was conducted for the classified satellite images of 2000, 2015, and 2025 using the Semi Automatic Classification Plugin (SCP) in QGIS. Validation data derived from high-resolution Google Earth imagery and field observations confirmed the reliability of classification results. The overall accuracies exceeded 0.88, with Kappa coefficients ranging from 0.83 to 0.85 (Table 1), indicating strong agreement between the classified and reference datasets. Minor confusion occurred between built-up and bare-land classes due to their similar spectral reflectance in arid urban settings, while vegetation achieved the highest accuracies owing to their distinct spectral signatures in the near-infrared and shortwave infrared bands.

**Table 1:** Accuracy assessment results for land-use/land-cover (LULC) classification of King Saud Medical City, 2000–2025.

Year	Built-up UA (%)	Vegetation UA (%)	Bare land UA (%)	Overall accuracy (%)	Kappa coefficient (*κ*)
2000	83.0	89.4	80.6	87.1	0.83
2015	86.5	88.0	82.7	88.4	0.84
2025	90.2	85.5	81.3	88.7	0.85

**Figure 1:**
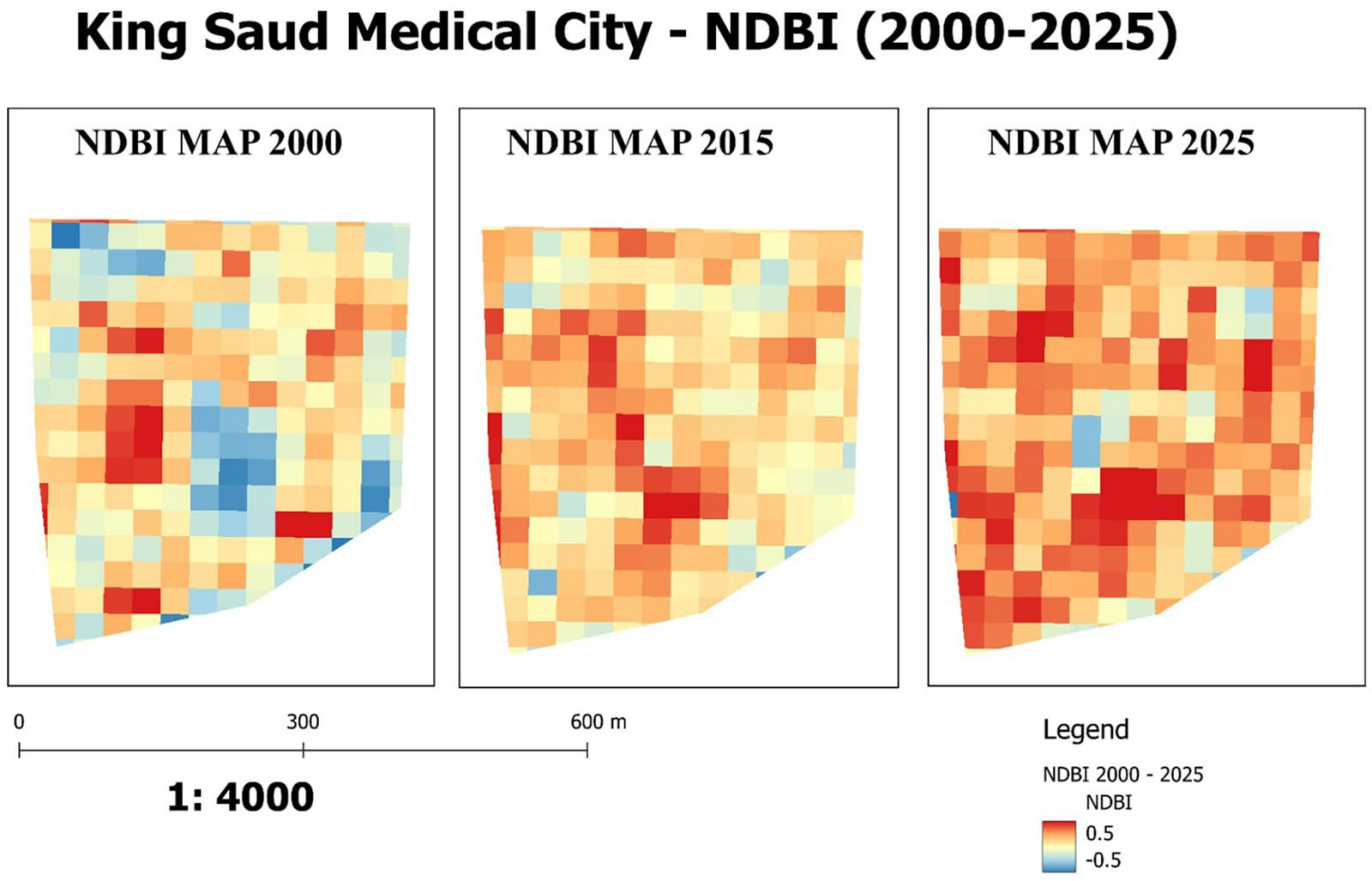
Land use changes at King Saud Medical City (2000–2025). showing reduced vegetation and bare land alongside a sharp increase in built-up areas.

These accuracy statistics validate the soundness of the following LULC change analysis so that all transition relationships established are based on dependable classification results. The matrices for detecting changes disclosed the magnitude and direction of transitions between land-use types. Between 2000 and 2025, areas of vegetation (1.89 ha) and barren land (7.19 ha) were transformed to built-up lands as well. Small-scale reversals between built-up and bare surfaces were also detected, portraying the partial irreversibility of urban growth once it had started. Taken together, the findings draw attention to a systematic one-way development of campus land uses where built-up areas expand at the cost of ecologically significant buffers and open space. The pattern of built-up intensity is presented in the NDBI maps for 2015 and 2025 (Figure 1). The constructed patches in 2015 were seemingly scattered and discretized in pattern, resulting in low and median density clusters of vegetation and barren land patches. In 2025, positive NDBI values were increasing, particularly in the central and northeastern areas of the campus. These hotspots coincide with the construction of multi-storey podia, as well as clinical and administrative buildings, reflecting vertical densification of the built-up area. Collectively, these hotspots indicate upward densification of the hospital complex alongside a progressive reduction of the green belt.

Spatial analysis of built-up intensity through the Normalized Difference Built-up Index (NDBI) further illustrates the transformation of the campus environment. In 2015, NDBI values indicated scattered, medium- to low-density built-up clusters distributed unevenly across the medical city, interspersed with pockets of bare land and vegetation. By 2025, however, the NDBI maps revealed a marked rise in positive index values, concentrated mainly in the central and northeastern sectors of the campus. These emerging high-density zones correspond to the construction of new multi-story clinical and administrative towers that replaced what were once fragmented, horizontally oriented structures. Simultaneously, negative NDBI values in peripheral areas diminished, reflecting the reduction of non-built-up surfaces and further emphasizing the trend of accelerated urban consolidation. The NDBI analysis was initiated in 2015, coinciding with the availability of Sentinel imagery at 10 m spatial resolution, which provided finer accuracy for the relatively small study area compared to the coarser 30 m Landsat imagery available for earlier years. Although Landsat data exist for 2000, their larger pixel size would have limited the reliability of index-based analysis; therefore, Sentinel data were selected for the NDBI mapping.

**Figure 2:**
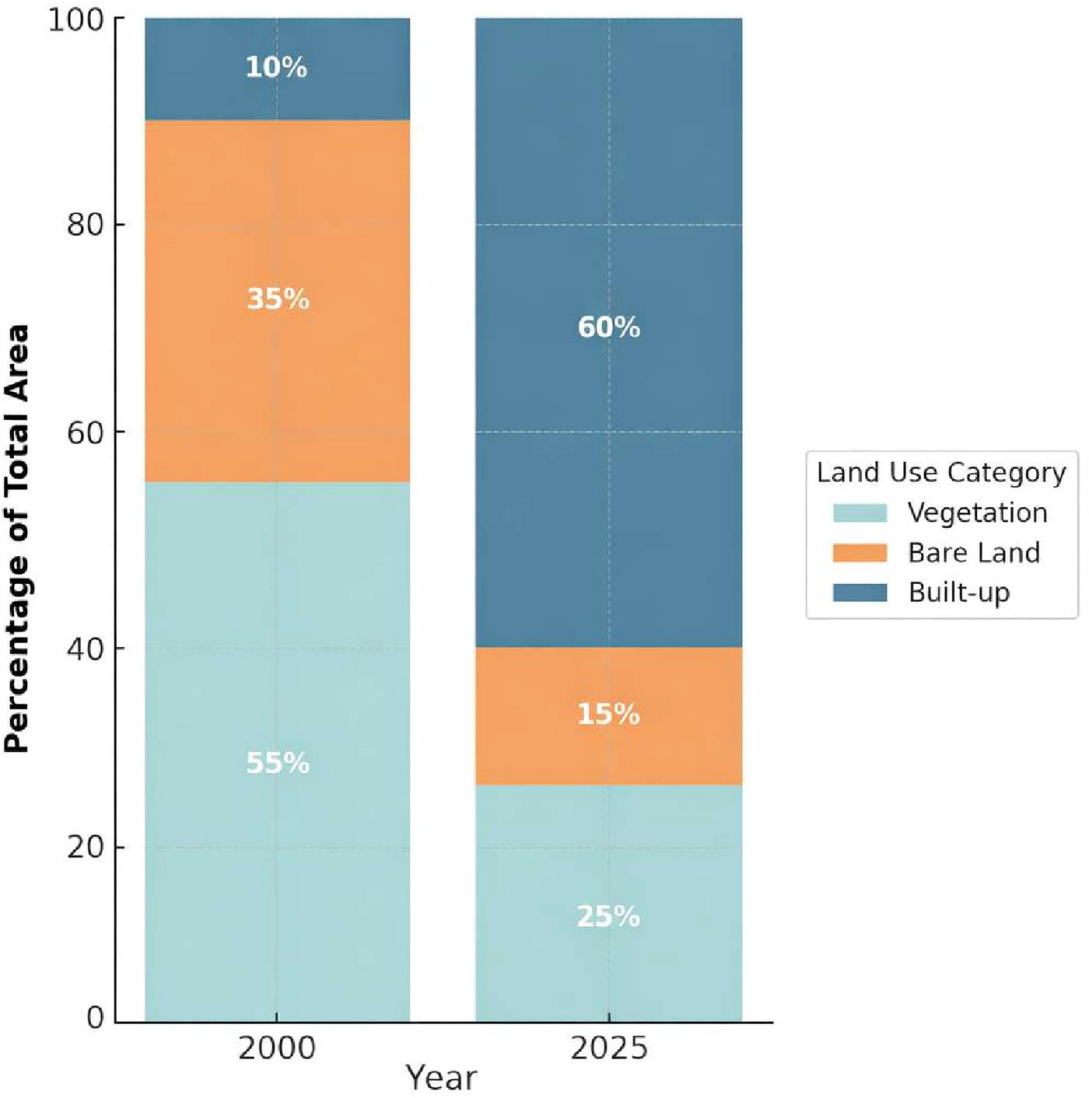
Land use distribution at King Saud Medical City in 2000 and 2025.

Complementary evidence is provided in the grouped bar chart (Figure 2), which depicts proportional land-use distributions in 2000 vs. 2025. Vegetation declined sharply from 55% to 25% of the total area, while bare land decreased from 35% to 15%. The land area under built-up increased by a multiple of six from 10% to 60% of the total area. These numbers also depict the transformation of a horizontally disjointed campus full of open and green space to an organized, solidly built-up medical campus. The figure draws attention to the extent and price of this expansion in terms of ecological buffers.

Collectively, these findings indicate the spatial and temporal changes were aligned with substantial enhancement of health care capacity. By 2025 KSMC has grown to be more than three times the size it was in 2000, comprising around 1,400 beds (21), of which about 200 are for ICU. It would also launch nine digital and robotic operating theaters by 2020 to do almost 19,000 major surgeries in a year. Emergency department grew to 102 beds, becoming the largest of its kind in Saudi Arabia. These infrastructural upgrades parallel Riyadh's rapid demographic growth from 3.6 million residents in 2000 to nearly 7.95 million in 2025 demonstrating how population pressure directly fueled hospital densification and modernization.

### 3.1 King Saud Medical City: development and statistics

As KSMC is one of the oldest and largest Ministry of Health facilities in the country (14, 15). It provides comprehensive medical services across all specialties, and its emergency departments (general, obstetrics, and pediatrics) together handle roughly half a million cases per year (16). The hospital's surgical workload is also very high on the order of ∼18,000 major operations performed annually (excluding open-heart surgeries) in recent years (17). Such figures rank KSMC's emergency and surgical departments among the busiest in Saudi Arabia, reflecting its role as a major referral center in the region (18). This ongoing expansion and modernization of the infrastructure, equipment, and clinical programs at KSMC have aimed at it keeping pace with leading hospitals globally, in line with Saudi's health sector development goals.

### 3.2 Patient volume and surgical statistics (1999–2001)

Official annual statistics from the early 1420s Hijri (circa 2000–2002) illustrate the enormous volume of patients served by KSMC instead of Riyadh Medical Complex. In 1999–2000, the complex received 1,071,224 outpatient visits and emergency cases in total and conducted 10,560 major surgical operations, with 11,671 newborn deliveries recorded that year, and in 2001–2002, KSMC treated 1,037,571 patients, and the number of major surgical procedures jumped to 32,576, in addition to 11,893 infants born at the facility that year (19). Taken together, the spatial analysis and service statistics confirm that the expansion of King Saud Medical City was not merely a matter of physical construction but a direct institutional response to rising healthcare demand. Historical records already documented patient volumes exceeding one million annually and surgical procedures surpassing 30,000 cases by the early 2000s (19). By 2025, these pressures translated into a tripling of bed capacity, the scaling of intensive care units, and the establishment of the Kingdom's largest emergency department (17). This alignment between geospatial evidence and institutional statistics underscores that spatial densification at KSMC was both a reflection of demographic and epidemiological pressures and a policy-driven modernization.

## 4 Discussion

Spatial expansion was therefore interpreted within reform phases rather than merely as chronological growth, enabling policy contextualized interpretation instead of purely descriptive land use change analysis.

The findings of this study reveal that King Saud Medical City underwent a profound physical transformation between 2000 and 2025, shifting from a fragmented campus of low-rise buildings into a consolidated vertical complex. This transformation of the built environment corresponded with escalating service demand and institutional scaling, and aligns with a growing international pattern in which large medical cities move toward vertical densification and service co-location in land-constrained environments. Remote sensing evidence, with consistently high classification accuracy (0.88–0.89 overall accuracy; *κ* 0.83–0.85), strengthens the reliability of these spatial observations, demonstrating more than 19 hectares of built-up expansion at the expense of bare land and vegetation.

Beyond descriptive growth, the proportional relationship between spatial expansion and institutional scaling is notable. Built-up land increased from 10% to 60% of total campus area (a sixfold rise), while bed capacity increased approximately threefold over the same period. Similarly, the introduction of 200 ICU beds and nine digital and robotic operating theaters by 2020 coincided temporally with the highest phase of built-up densification. Although this temporal alignment suggests structural coherence between spatial consolidation and service expansion, causality cannot be definitively established within the limits of retrospective observational analysis. Rather, the findings indicate a strong institutional spatial correspondence, in which physical densification appears to have enabled increased functional capacity. At the same time, this transformation highlights the interplay between demographic pressures and policy frameworks. Riyadh's population nearly doubled during the study period, amplifying demand for acute care services and rendering earlier horizontal configurations increasingly inefficient. National reforms, including the 2014 Medical Cities Law, the clustering model, and Vision 2030's focus on health system modernization provided an enabling institutional environment for KSMC to scale capacity rapidly. In this study, these reforms are interpreted as enabling institutional scaling rather than as direct causal drivers of spatial expansion. They functioned as governance frameworks that facilitated infrastructure modernization, digital integration, and capacity consolidation. The near-total conversion of open and vegetated spaces suggests potential sustainability trade-offs, although no quantitative environmental indicators were assessed in this study. However, these observations should be interpreted cautiously. The findings suggest potential trade-offs between densification efficiency and ecological buffering, indicating that future expansions may benefit from embedding modular design and green infrastructure strategies within hospital master planning. Collectively, these results illustrate both the opportunities and constraints of hospital growth in rapidly urbanizing cities. Spatial densification supported service scaling and modernization, yet it also reduced ecological buffers. The integration of geospatial monitoring with institutional metrics offers a structured approach to balancing efficiency, capacity growth, and environmental considerations in future health infrastructure planning.

### 4.1 Transferability and external validity

The spatial–temporal dynamics documented in KSMC resonate with international patterns of hospital densification observed in land-scarce urban settings. Although this research analyzed a single institutional case, its methodological framework linking remote sensing–derived built-up indices with institutional performance metrics proposes a conceptual analytical framework that requires further cross-case validation. Comparable studies in Singapore, Tokyo, and Seoul demonstrate how vertical hospital design and spatial consolidation have enhanced service efficiency and adaptive capacity in constrained urban environments (9, 12, 14).

Importantly, the present study does not claim universal generalizability but rather proposes a conceptual analytical template that may be tested across additional contexts. Built-form intensification appears to co-evolve with organizational scaling and diversification of clinical services; however, the strength and direction of this relationship may be moderated by regulatory density limits, financing structures, transport connectivity, and climatic constraints. Future cross-city analyses across Gulf and Asian megacities could empirically validate these pathways and refine the institutional–spatial proportionality model proposed here.

### 4.2 Policy implications

The transformation of King Saud Medical City over the past two decades reflects not only physical expansion but also the broader institutional evolution of Saudi Arabia's tertiary healthcare system. The rapid densification and capacity increase revealed by this study underscore the importance of integrating spatial intelligence with health service planning to achieve balanced and resilient delivery models.

At the system level, policy frameworks may consider embedding evidence-based service planning in which spatial growth indicators are interpreted alongside hospital utilization rates, referral patterns, and population health data. Such integration would support alignment between infrastructure development and actual service demand, reducing the risk of reactive or project-driven expansion. This perspective is consistent with the Health Transformation Program under Vision 2030, which prioritizes equitable access, efficiency, and value-based healthcare (7, 9).

However, it is important to distinguish between empirical findings and normative recommendations. The present study empirically demonstrates spatial–institutional alignment; it does not directly evaluate governance effectiveness or policy performance outcomes. Therefore, suggested planning strategies should be understood as informed implications rather than as prescriptive conclusions derived from causal testing.

Establishing national benchmarks for hospital catchment areas, bed-to-population ratios, and functional integration between tertiary and primary care facilities could enhance coherence within the evolving health cluster system. At the governance level, collaboration between the Ministry of Health, academic institutions, and regional health clusters may facilitate coordinated investment decisions supported by geospatial intelligence platforms capable of dynamically monitoring service coverage and emergency accessibility (20).

Adopting a learning health system approach where institutional performance data, patient outcomes, and spatial indicators are continuously integrated into planning cycles may further strengthen adaptive capacity. In this context, KSMC may serve as an illustrative case of how spatial evidence can inform institutional scaling, while acknowledging that governance optimization requires additional evaluative research. Geospatial monitoring may also be used prospectively to establish threshold indicators for vertical expansion and green space preservation, supporting proactive planning rather than reactive infrastructure growth.

## 5 Conclusion

This study has provided a novel spatial–temporal analysis of King Saud Medical City (KSMC) between 2000 and 2025, demonstrating how the hospital transitioned from a horizontally fragmented campus into a vertically consolidated medical complex. By answering the core research questions, the analysis revealed the spatial pattern of built-up expansion, the institutional dynamics driving land-use transitions, and the policy lessons necessary for guiding future medical city planning in Saudi Arabia.

The key innovation of this work lies in being the first study to employ remote sensing and GIS evidence to examine the long-term evolution of a major Saudi hospital, linking geospatial change directly with healthcare capacity and service provision. This approach moves beyond traditional health system assessments to show how the built environment encodes demographic pressures, institutional growth, and policy reforms under frameworks such as Vision 2030.

Importantly, the findings highlight both opportunities and trade-offs: while capacity expanded threefold and service delivery improved, this growth came at the cost of ecological buffers and open spaces. Embedding geospatial evidence into health infrastructure governance may support more informed planning decisions and contribute to balanced infrastructure expansion.

Looking forward, future research could extend this methodology to other medical cities across the Kingdom or develop predictive geospatial models to anticipate how medical cities might expand under different demographic and policy scenarios. Such work may contribute to the development of an evidence base for sustainable, spatially informed healthcare planning in rapidly urbanizing contexts.

## Data Availability

Publicly available datasets were analyzed in this study. This data can be found here: N/A.

## Appendix:

Accuracy Metrics:

**Table:**

Metric	Formula	Result (%)	Description
Overall accuracy (OA)	(Correctly classified samples/Total samples) × 100	88.0	Indicates the proportion of correctly classified samples.
Producer's accuracy (PA)	Correctly classified per class/Total reference for that class × 100	Built-up: 87.3; Vegetation: 86.2; Bare Land: 83.3	Measures the probability that a reference pixel was correctly classified.
User's accuracy (UA)	Correctly classified per class/Total classified samples for that class × 100	Built-up: 87.3; Vegetation: 86.2; Bare Land: 83.3	Indicates the reliability of each class.
Kappa coefficient (κ)	κ = (po - pe)/(1 - pe)	0.85	Represents the level of agreement between classification and reference data beyond chance.
